# Mental Health among Adults during the COVID-19 Pandemic Lockdown: A Cross-Sectional Multi-Country Comparison

**DOI:** 10.3390/ijerph18052686

**Published:** 2021-03-07

**Authors:** Kele Ding, Jingzhen Yang, Ming-Kai Chin, Lindsay Sullivan, Giyasettin Demirhan, Veronica Violant-Holz, Ricardo R. Uvinha, Jianhui Dai, Xia Xu, Biljana Popeska, Zornitza Mladenova, Waheeda Khan, Garry Kuan, Govindasamy Balasekaran, Gary A. Smith, on behalf of Global Community Health–COVID-19 Collaborative Research Team

**Affiliations:** 1 School of Health Science, College of Education Health & Human Service, Kent State University, Kent, OH 44242, USA; kding@kent.edu; 2 Center for Injury Research and Policy, The Abigail Wexner Research Institute at Nationwide Children’s Hospital, Columbus, OH 43205, USA; Gary.Smith@nationwidechildrens.org; 3 Foundation for Global Community Health, Las Vegas, NV 89012, USA; 4 Discipline of Children’s Studies, School of Education, National University of Ireland, H91 Galway, Ireland; lindsaymarie.sullivan@nuigalway.ie; 5 Department of Physical Education and Sport Teaching, Faculty of Sport Sciences, Hacettepe University, Ankara 06420, Turkey; demirhan@hacettepe.edu.tr; 6 Department of Didactics and Educative Organization, University of Barcelona, 08015 Barcelona, Spain; vviolant@ub.edu; 7 School of Arts, Sciences and Humanities, University of Sao Paulo, Sao Paulo 01310, Brazil; uvinha@usp.br; 8 School of Physical Education and Sports, Soochow University, Suzhou 215021, China; sddjh@suda.edu.cn; 9 Hubei Key Laboratory of Sport Training and Monitoring, Wuhan Sports University, Wuhan 430021, China; xuxia@whsu.edu.cn; 10 Faculty of Educational Sciences, Goce Delcev University, 2000 Stip, North Macedonia; biljana.popeska@ugd.edu.mk; 11 Association of Touristic Animators, 1000 Sofia, Bulgaria; z.mladenovaz@gmail.com; 12 Faculty of Behavioural Sciences, SGT University, Gurugram 122505, India; profwkhan@gmail.com; 13 Exercise and Sports Science, School of Health Sciences, Universiti Sains Malaysia, Kubang Kerian 16150, Malaysia; garry@usm.my; 14 National Institute of Education, Nanyang Technological University, Singapore 178957, Singapore; govindasamy.b@nie.edu.sg

**Keywords:** COVID-19, mental health, anxiety, depression, multi-country, resilient coping, hope, adults

## Abstract

Despite the global impact of COVID-19, studies comparing the effects of COVID-19 on population mental health across countries are sparse. This study aimed to compare anxiety and depression symptoms during the COVID-19 lockdown among adults from 11 countries and to examine their associations with country-level COVID-19 factors and personal COVID-19 exposure. A cross-sectional survey was conducted among adults (≥18 years) in 11 countries (Brazil, Bulgaria, China, India, Ireland, North Macedonia, Malaysia, Singapore, Spain, Turkey, United States). Mental health (anxiety, depression, resilient coping, hope) and other study data were collected between June–August 2020. Of the 13,263 participants, 62.8% were female and 51.7% were 18–34 years old. Participants living in Brazil had the highest anxiety and depression symptoms while participants living in Singapore had the lowest. Greater personal COVID-19 exposure was associated with increased anxiety and depression symptoms, but country-level COVID-19 factors were not. Higher levels of hope were associated with reduced anxiety and depression; higher levels of resilient coping were associated with reduced anxiety but not depression. Substantial variations exist in anxiety and depression symptoms across countries during the COVID-19 lockdown, with personal COVID-19 exposure being a significant risk factor. Strategies that mitigate COVID-19 exposure and enhance hope and resilience may reduce anxiety and depression during global emergencies.

## 1. Introduction

On 11 March 2020, the World Health Organization declared the novel coronavirus (COVID-19) outbreak a global pandemic [1]. Governments worldwide imposed “lockdown,” “stay-at-home,” and “quarantine” policies and restrictions, most of which were in effect for weeks to months [2]. Although these public health measures were critical to slow the spread of COVID-19, the first major pandemic of our generation, these measures forced people worldwide to adapt to a “new normal” way of life [2,3]. This new normal, coupled with the risk of being infected with a potentially life-threatening virus, resulted in vast disruptions to the daily lives of people of all ages in countries around the world [4,5,6].

Recently published studies found that the COVID-19 pandemic negatively impacted people’s mental health and well-being, leading to increased feelings of social isolation and loneliness [7,8], and increased psychological distress, fear, anxiety, and depression [9,10,11,12,13]. The long-term effects of COVID-19 are unknown, although experts suggest that the pandemic may have long-lasting physical and mental health consequences [14]. Evidence suggests female sex, younger age, having a diagnosed mental disorder, or being a healthcare worker are risk factors for negative mental health outcomes during the COVID-19 pandemic [5,6,7,8,9,10,15,16]. Additional evidence suggests that governmental policies varied in timing and stringency across countries, leading to disproportionate effects on different communities and possible mental health disparities [6,7,8,14]. Despite the global impact of COVID-19, most published studies are limited by the collection of data from only one country. No published study to date has examined population mental health during the COVID-19 lockdown among the general adult population across multiple countries from around the world [6,9]. 

This study compared anxiety and depression symptoms during the COVID-19 lockdown among adults ≥18 years old, who resided in one of 11 countries. Specifically, we examined whether country-level COVID-19 factors (i.e., increase in confirmed cases per million people and governmental policy response) and personal COVID-19 exposure (i.e., had close contact with someone with COVID-19) were associated with anxiety and depression symptoms during lockdown and whether a person’s resilient coping and hope were associated with reduced anxiety and depression symptoms. Our findings will provide a summary of mental health outcomes including anxiety, depression symptoms, resilient coping, and hope, during the COVID-19 lockdown among adults from 11 counties. These findings may help us begin to understand the associations between country- and personal-level factors and mental health during the COVID-19 global pandemic.

## 2. Materials and Methods

### 2.1. Study Design

We conducted a cross-sectional, multi-country survey study in 11 countries (Brazil, China, India, Ireland, North Macedonia, Malaysia, Singapore, Slovakia, Spain, Turkey, USA). Participants were a convenience sample of adult residents (≥18 years old) recruited from one of the 11 countries (Table 1). Before survey distribution, we established a collaborative research team in each of the participating countries. Each team comprised a designated leader and three to five members. The US team led the development of the study protocol and coordinated all study activities. The other teams were responsible for implementing research activities within their respective country following the established study protocol. The study first received ethical approval on 22 May 2020 from the Institutional Review Board (IRB) at Nationwide Children’s Hospital (in the US, IRB ID = STUDY00001110) and then received ethical approval from the respective IRB at each participating institution in the other countries. Patients or the public were not involved in the design, or conduct, or reporting, or dissemination plans of our research.

### 2.2. Survey Instrument

The US team developed the initial survey instrument in English based on validated instruments [17,18,19] and published studies on the mental health impact of the COVID-19 pandemic [4,5,6,7,8,9]. We sent the initial instrument, along with written instructions, to each team for expert review. We revised the instrument based on the feedback received from each team and then returned it to the teams for further consideration. This process was repeated until the final draft of the instrument was agreed upon by all teams. We then pilot-tested the instrument in all 11 countries (*n* = 131 total returned surveys) and updated the instrument based on the results. Lastly, we solicited additional feedback from the team leaders before finalizing the English version of the instrument. The final survey instrument included 73 questions, including skip-pattern questions.

Seven countries used non-English versions of the instrument. These countries translated the final instrument into their country’s official language and back-translated it into English to ensure accuracy. Independent bilingual professionals who were not team members performed the translations. Cultural relevance was considered during the translation. For example, the translated response categories of income levels were based on the quartile of the income in the country rather than direct conversion of US dollars. This study used eight languages: Bulgarian, simplified Chinese, English, North Macedonian, Malay, Portuguese, Spanish, and Turkish.

### 2.3. Procedures

Team leaders sent invitations, including a link for an anonymous online survey (e.g., Qualtrics) to potential participants via the study flyer posted on both their personal and institution’s social media accounts (e.g., Facebook, Twitter, WeChat). The first page of the survey contained a screening question to determine participant eligibility (≥18 years old). Eligible participants were directed to review study information, including instructions on voluntary informed consent before proceeding to the survey; otherwise, participation was discontinued. The survey took approximately 15 min to complete. Data were collected between 11 June 2020, and 31 August 2020. Each country collected data for approximately 30 days, with varying start dates. Following data collection, each team downloaded their country’s survey data and securely sent it to the US team for management and analysis. We received 15,518 surveys from 11 countries. Of these, 2255 (14.5%) surveys were excluded because of missing data. The final sample for this study included 13,263 surveys.

### 2.4. Study Variables and Measures

*Anxiety and depression* were measured using the established, empirically validated Adult Patient-Reported Outcomes Measurement Information System (PROMIS^®^) Short Form v1.0–Anxiety 4a (Cronbach’s alpha of 0.93) and PROMIS^®^ Short Form v1.0–Depression 4a (Cronbach’s alpha of 0.95) [17]. Both scales, originally developed for PROMIS^®^, are available in >40 languages, and can be used for both the general population and for persons living with chronic conditions [18]. Each scale included four questions on a 5-category Likert-scale (range = 4 to 20) with higher scores representing more anxiety (or depression) symptoms. We asked participants to respond to the questions based on their feelings and thoughts during their country’s lockdown. For data analysis, we converted the original total raw scores to T-scores [17]. The PROMIS^®^ Health Organization provided us with each of the seven non-English versions of the two forms, the translation licenses, and the certified translations [18].

*Resilient coping* was measured using the 4-item Brief Resilient Coping Scale, a 5-category symmetrical Likert-scale with a Cronbach’s alpha of 0.76 and test-retest reliability of 0.71 [19]. We asked participants to respond to the questions based on their feelings during their country’s lockdown. As recommended [19], the sum of response scores (range = 4 to 20) was used in the analysis, with higher scores indicating a higher level of resilient coping.

*Hope* was measured using the 12-item Herth Hope Index, a valid scale that is used in various languages across many countries, with a Cronbach’s alpha of 0.97 and test-retest reliability of 0.91 [20]. We asked participants about their desire for and belief in a positive future during the lockdown period. Participants rated their agreement with each item on a 4-point Likert-scale from *strongly disagree* to *strongly agree*. A total hope score (range = 12 to 48) was calculated and used for data analysis, with higher scores indicating a higher level of hope [20].

*Country-level COVID-19 factors* were measured using two variables, based on existing data retrieved from the online resource [21]. *The increase in confirmed cases* per million people was calculated as the change in the cumulative number of confirmed COVID-19 cases per million people in a country between the date the participating country reached its 100th case and 31 August 2020 (the last day of data collection), divided by the number of days elapsed between these two dates. *Governmental policy response* to COVID-19 was determined by the Government Stringency Index (GSI), a daily composite score covering nine policy areas (e.g., school closures, travel bans, contact tracing) and rescaled to a value from 0 to 100 (0 = no policy response, 100 = strictest policy responses) [22]. We used the average scores between the two dates described above to represent strictness of governmental policy response to COVID-19.

*Personal COVID-19 exposure* was measured using eight questions that asked participants about their COVID-19-related exposures during their country’s lockdown. We created a single variable to capture each participant’s highest COVID-19 risk exposure based on their responses. We classified each participant into one of five mutually exclusive categories (5 = high-risk exposure, 1 = low-risk exposure) based on each participant’s highest COVID-19 exposure. A score of 5 indicated a participant had been diagnosed with COVID-19 or had COVID-19 symptoms; 4–participant had close contact with someone with COVID-19 or had to quarantine for 14 days; 3–participant went out (i.e., left house or property) at least once a week for work-related or personal activities; 2–participant went out at least once a month (but less than once a week) for work-related or personal activities; 1–participant went out less than once a month during lockdown.

*Demographic variables* included sex, age group, highest level of education, marital status, employment and income changes since the start of the COVID-19 pandemic, the number of people co-habiting during the lockdown, and history of a mental disorder.

### 2.5. Statistical Analysis

We conducted descriptive analyses of demographic, country-level COVID-19 factors, personal COVID-19 exposure, and mental health variables (anxiety, depression, resilient coping, hope). We assessed the zero-order correlations among the country-level COVID-19 factors, personal COVID-19 exposure, and mental health variables. We examined differences in the mental health variables across the countries using ANOVA tests. We analyzed the associations of country-level COVID-19 factors and personal COVID-19 exposure with anxiety and depression symptoms using two-level, linear mixed models in a four-model testing process.

Before the model testing, we evaluated the normal distribution of multivariate residuals of anxiety and depression scores and identified outliers using Jackknife statistics. For model testing, we treated the participants (level 1) as clusters within their countries (level 2) to account for correlations among the participants from the same country. We used a random intercept test for variance estimation accuracy. Model 1 was a null model with no fixed factors but a random intercept of the country. For Model 2, we added country-level COVID-19 factors and personal COVID-19 exposure variables as fixed factors. For Model 3, we added resilient coping and hope. Model 4 adjusted for demographic variables, including sex, age, educational level, marital status, employment and/or income changes since the start of the COVID-19 pandemic, the number of people co-habiting during lockdown, and history of a mental disorder. We used intra-level correlation coefficients to evaluate the need for random effect control and Akaike information criterion and Bayesian information criterion to determine the model fit and improvement sequentially from Models 1 through 4 [23]. We conducted data analyses between 1 September 2020, and 31 October 2020, using SAS version 9.4. We set the significance level at α = 0.05.

## 3. Results

### 3.1. Study Participants

Of the 13,263 participants included, 62.8% were female (*n* = 8332), 51.7% were 18–34 years old (*n* = 6857), 60.4% had a bachelor’s degree or higher (*n* = 8002), 48.7% were single (*n* = 6465), and 15.3% (*n* = 2022) had a history of a mental disorder (Table 1). China had the greatest number of participants (*n* = 2850, 21.5%), with 1189 (9.0%) from Wuhan, where the first COVID-19 case was identified, followed by Brazil (*n* = 1500, 11.3%) and Turkey (*n* = 1400, 10.6%).

### 3.2. Country-Level COVID-19 Factors and Personal COVID-19 Exposure

**Figure 1:**
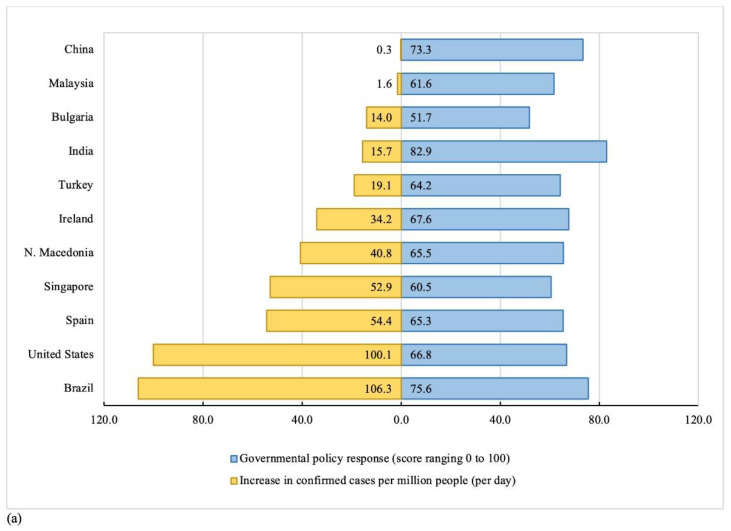

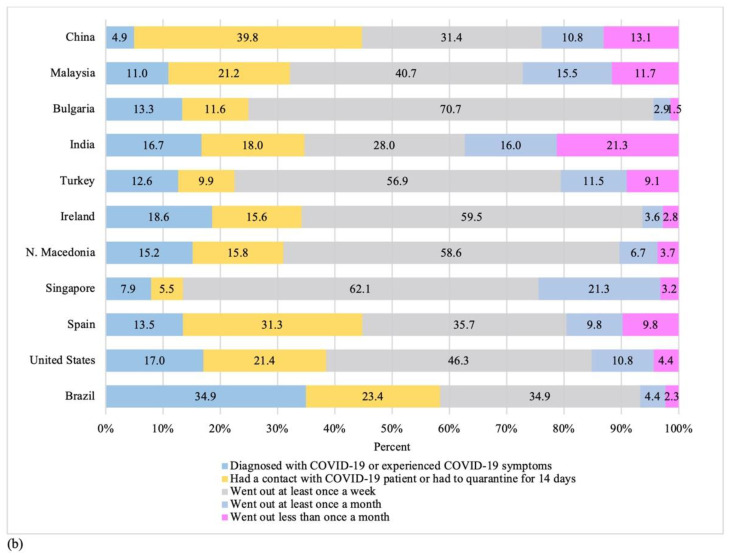
Distribution of country-level COVID-19 factors and personal COVID-19 exposures by participating countries: (**a**) Country-level COVID-19 factors; (**b**) Personal COVID-19 exposures. Note: Country-level COVID-19 factors were measured using two variables: *Increase in confirmed cases per million people,* capturing each participating country’s average confirmed COVID-19 cases per million people per day during their lockdown; and *Governmental policy response* to COVID-19, capturing each participating country’s average government stringency index score during their lockdown (0 = no policy response, 100 = strictest policy responses). Government stringency index score was not available for North Macedonia, thus, we used the average scores of its 5 neighboring countries (Albania, Bulgaria, Greece, Kosovo, Serbia) instead. Personal COVID-19 exposure was created based on participants’ COVID-19-related exposures during their country’s lockdown, capturing their highest risk exposure in 1 of 5 mutually exclusive categories (5 = high-risk exposure, 1 = low-risk exposure).

The average daily increase in confirmed COVID-19 cases per million people varied substantially across countries (Figure 1a). Brazil (106.3 cases/million people) and the United States (100.1 cases/million people) experienced the greatest increase in confirmed COVID-19 cases during the study period. China (0.3 cases/million people) and Malaysia (1.6 cases/million people) had the lowest increase in confirmed COVID-19 cases. India, Brazil, and China had the most stringent governmental policy responses with average daily GSI scores of 82.9, 75.6, and 73.3, respectively (Figure 1a). Bulgaria had the lowest GSI score (51.7).

Of the 13,263 participants, 12.5% experienced COVID-19 symptoms, while only 1.9% received a confirmed COVID-19 diagnosis. Approximately one quarter (22.2%) of participants had close contact with someone with COVID-19 or had to quarantine for 14 days (Table 1). We observed significant variations in proportions of personal COVID-19 exposure across the countries (*p* < *0*.0001) (Figure 1b). Participants living in Brazil reported the highest proportion (34.9%) of confirmed COVID-19 diagnoses or COVID-19 symptoms. Participants living in China reported the highest proportion (39.8%) of having had close contact with someone with COVID-19 or having had to quarantine for 14 days, followed by participants who lived in Spain (31.3%) or Brazil (23.4%).

### 3.3. Mental Health across the 11 Countries

**Figure 2:**
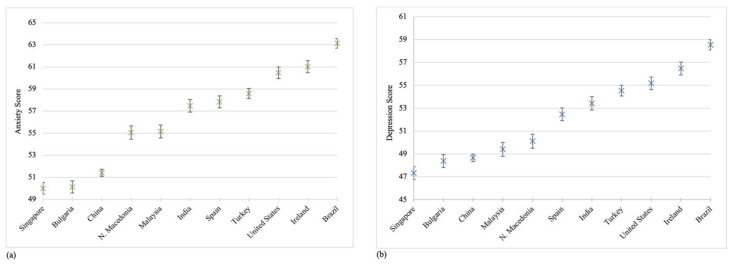

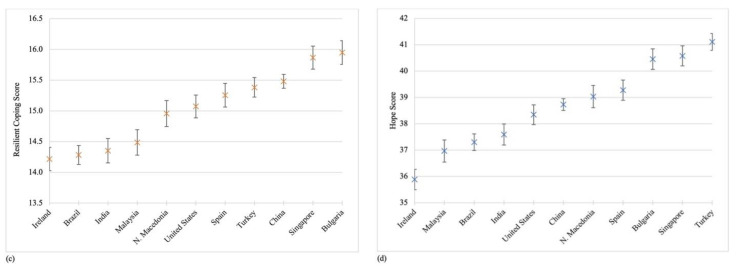
Mean scores of four mental health variables by participating countries: (**a**) Anxiety; (**b**) Depression; (**c**) Resilient Coping; (**d**) Hope. Note: *p*-values were based on Bonferroni post hoc tests accounting for multiple comparison following ANOVA. T-Score was used in the calculation of mean scores for anxiety and depression symptoms. The mean scores of four mental health variables (anxiety, depression, resilient coping, and hope) are displayed, along with 95%CIs, for each county from low to high. We only describe the statistically significant results when a country was compared to other countries with higher scores. *Anxiety.* We found statistically significant differences in mean anxiety scores when we conducted the following comparisons: (1) Singapore with all other countries, with the exception of Bulgaria; (2) Bulgaria with all other countries; (3) China with all other countries; (4) North Macedonia with all other countries, with the exception of Malaysia; (5) Malaysia with all other countries; (6) India with all other countries, with the exceptions of Spain and Turkey; (7) Spain with all other countries, with the exception of Turkey; (8) Turkey with all other countries; (9) United States with Brazil; and (10) Ireland with Brazil. *Depression.* We found statistically significant differences in mean depression scores when we conducted the following comparisons: (1) Singapore with all other countries, with the exception of Bulgaria; (2) Bulgaria with all other countries, with the exceptions of China and Malaysia; (3) China with all other countries, with the exceptions of Malaysia and North Macedonia; (4) Malaysia with all other countries, with the exception of North Macedonia; (5) North Macedonia with all other countries; (6) Spain with all other countries, with the exception of India; (7) India with all other countries, with the exception of Turkey; (8) Turkey with all other countries, with the exception of United States; (9) United States with Brazil; and (10) Ireland with Brazil. *Resilient coping.* We found statistically significant differences in mean resilient coping scores when we conducted the following comparisons: (1) Ireland with all other countries, with the exceptions of Brazil, India and Malaysia; (2) Brazil with all other countries, with the exceptions of India and Malaysia; (3) India with all other countries, with the exception of Malaysia; (4) Malaysia with all other countries; (5) North Macedonia with all other countries, with the exceptions of United States and Spain; (6) United States with all other countries, with the exception of Spain; (7) Spain with all other countries; (8) Turkey with all other countries; and, (9) China with Singapore and Bulgaria. *Hope.* We found statistically significant differences in mean hope scores when we conducted the following comparisons: (1) Ireland with all other countries; (2) Malaysia with all other countries, with the exceptions of Brazil and India; (3) Brazil with all other countries, with the exception of India; (4) India with all other countries, with the exception of United States; (5) United States with all other countries, with the exceptions of China and North Macedonia; (6) China with all other countries, with the exceptions of North Macedonia and Spain; (7) North Macedonia with all other countries, with the exception of Spain; (8) Spain with all other countries; and (9) Bulgaria with Turkey.

The overall mean scores [Standard Deviation (SD)] of anxiety, depression, resilient coping, and hope were 56.1 (9.8), 52.1 (9.7), 15.1 (3.1), and 38.7 (6.3), respectively. Participants living in Brazil had the highest anxiety and depression scores while participants in Singapore had the lowest (Figure 2a,b). Participants living in Bulgaria reported the highest mean resilient coping scores [16.0 (SD = 3.3)] (Figure 2c), and participants living in Turkey reported the highest mean hope scores [41.1 (5.5)] (Figure 2d). Post hoc multiple comparison tests found significant differences in the mean anxiety, depression, resilient coping, and hope scores across the 11 countries.

### 3.4. Associations of Country-Level COVID-19 Factors and Personal COVID-19 Exposure with Anxiety and Depression Symptoms

The zero-order correlation tests showed significant, although weak, positive correlations between the country-level COVID-19 factors, personal-level COVID-19 exposure, and anxiety and depression (Table 2). The results of the multilevel linear mixed model analysis are depicted in Table 3. Increased personal COVID-19 exposure, but not country-level COVID-19 factors, was significantly associated with increased anxiety and depression symptoms (Table 3, Model 2). Higher resilient coping and hope scores were associated with significant reductions in anxiety and depression symptoms (Table 3, Model 3).

In our full model (Table 3, Model 4), participants who went out at least once a week during the lockdown reported higher anxiety (β = 0.65, 95%CI = 0.15, 1.14) and depression symptoms (β = 1.03, 95%CI = 0.52, 1.54) than participants who went out less than once a month, as did participants who had close contact with someone with COVID-19 or had to quarantine for 14 days (anxiety: β = 1.35, 95%CI = 0.83, 1.86; depression: β = 1.33, 95%CI = 0.79, 1.86). Participants who had COVID-19 or had COVID-19 symptoms reported higher anxiety (β = 2.18, 95%CI = 1.61, 2.75) and depression symptoms (β = 2.16, 95%CI = 1.57, 2.75) than participants who went out less than once a month during the lockdown. Participants who went out at least once a month during the lockdown (β = 1.40, 95%CI = 0.79, 2.01) reported higher depression symptoms than persons who went out less than once a month. The associations between country-level COVID-19 factors and anxiety and depression symptoms were not significant.

We found that a higher resilient coping score was associated with a significant reduction in anxiety (β = −0.13, 95%CI = −0.18, −0.08), but not with a reduction in depression symptoms (β = −0.03, 95%CI = −0.09, 0.02) (Table 3, Model 4). A higher hope score was associated with reduced anxiety (β = −0.54, 95%CI = −0.57, −0.51) and depression symptoms (β = −0.61, 95%CI = −0.64, −0.58). Female sex, younger age, history of a mental disorder, and experiencing a change in employment or reduction in income since the start of the pandemic were associated with increased anxiety and depression symptoms during the lockdown. Participants who were single reported significantly lower anxiety symptoms than married participants (β = −0.60, 95%CI = −0.98, −0.23).

## 4. Discussion

This multi-country comparison study is the first to assess anxiety and depression symptoms during the COVID-19 lockdown among adults across 11 countries and examine the associations of country- and personal-level factors with anxiety and depression symptoms. We found differences in anxiety and depression symptoms during lockdown across the countries and that greater personal COVID-19 exposure was a significant risk factor for increased anxiety and depression symptoms during the lockdown. However, we found no association between country-level COVID-19 factors and anxiety and depression symptoms after adjusting for personal COVID-19 exposure. Higher levels of hope were associated with reduced anxiety and depression symptoms; higher levels of resilient coping were associated with reduced anxiety but not depression. Our findings add to the current literature on the effects of the COVID-19 lockdown on mental health across countries, underscoring the importance of minimizing personal exposure, which may reduce the percentage of adults who experience adverse mental health outcomes during this public health crisis [4,5,6]. Our findings, along with others [13,14,15,16,24], highlight the need and importance of developing and testing culturally appropriate and tailored interventions that promote positive mental health and increase resilient coping during global public health emergencies.

We found significant differences in country-level COVID-19 factors and mental health outcomes across the 11 countries. The average scores for anxiety and depression were highest among participants living in Brazil while participants living in China were among those with the lowest average scores. These observed differences may reflect existing differences in mental health outcomes across countries. According to the World Health Organization, Brazil was among the countries with the highest prevalence of anxiety and depression while China was among those with the lowest prevalence of anxiety and depression prior to the COVID-19 pandemic [25]. Additionally, Brazil experienced the most significant increase in confirmed cases per million people during the study period, which may exacerbate anxiety and depression symptoms, whereas China experienced the lowest case rate. It appears that the rigorous Chinese lockdown measurements may have helped slow the spread of COVID-19 in China [26]. Other countries that lifted lockdown restrictions as of late June 2020 (e.g., Brazil and the US), had more COVID-19 cases, even when the country had relatively high average governmental policy responses [27]. Other possible explanations for the observed differences between countries include cultural differences, mental health awareness and service availability in each country, and the timing, length, and stringency of governmental restrictions [6,7,8,14,26,27,28]. Most importantly, culture can impact the perception of and care-seeking for mental health problems. For example, cultural stigma may influence how people describe their symptoms, and if they choose to disclose their symptoms. Cultural factors may also influence personal decisions to seek treatment for mental health problems, which in turn, could impact population mental health [29].

Our results revealed that neither increases in confirmed cases per million people nor a more stringent governmental policy response was associated with increased anxiety and depression symptoms during the lockdown, after adjusting for personal risk factors. These findings may appear to be counter-intuitive, because the COVID-19 pandemic is a traumatic event, leading to increased trauma, stress, and fear [5,11]. Fear of becoming infected or fear of the socio-economic consequences of the pandemic may increase as the number of COVID-19 cases increase, resulting in increased anxiety and depression symptoms [9,10,11]. In addition, although stringent governmental policy responses such as lockdown and physical distancing have slowed the spread of COVID-19 [2,3], such interventions disrupted daily living. Prior research shows such public health measures have contributed to adverse psychological health outcomes [6,7,8,9,11,14,30]. The few studies that have directly assessed the effects of governmental policy responses on mental health outcomes have reported mixed findings. Our results support previous findings that the effects of the COVID-19 lockdown on mental health impairments may not be observed immediately but may pose a risk for future mental health symptoms and disorders [7,8,9,17,31]. Additionally, the increase in confirmed COVID-19 cases and governmental policy responses have dramatically altered individuals’ daily lives. This change, coupled with personal-level risk factors, may have influenced participants’ responses to the pandemic, which may explain the insignificant influences of country-level factors on anxiety and depression symptoms when adjusting for person-level factors. Further, the country-level factors in this study were based on actual rather than perceived policy responses, the latter being more likely to be associated with mental health outcomes [8,9]. Further studies are needed to examine the short- and long-term effects of governmental policy responses to COVID-19 on population mental health.

Consistent with prior studies [7,8,9,14,15,16], we found greater personal exposure to COVID-19, including going out multiple times a month or having close contact with a confirmed COVID-19 case, was associated with increased anxiety and depression symptoms during the lockdown. Previous studies found that the risk of depression was higher among patients with COVID-19 [16] and healthcare workers than the general population [5,15,32]. Lai et al. found that approximately half of the healthcare workers in China who were exposed to patients with COVID-19 reported symptoms of anxiety (44.6%) or depression (50.4%) [15]. Other studies also suggest that front-line healthcare workers are at risk of experiencing anxiety or depression symptoms during the COVID-19 pandemic [32,33]. Therefore, individuals who have a higher likelihood of COVID-19 exposure should have access to mental health and emotional support services. Preventive strategies and early interventions are required to minimize COVID-19 exposures and support COVID-19 survivors [14].

Mental health consequences of COVID-19 are unevenly distributed across populations. Consistent with prior studies [5,9,10,13], we found that anxiety and depression symptoms were magnified among vulnerable groups, including women, young adults, and individuals with a history of mental disorder. Our findings suggest that mental health interventions should target these vulnerable groups to help prevent the widening of health inequalities [33]. We also found that higher levels of resilient coping and hope were associated with reduced anxiety and depression symptoms, highlighting the potential protective effects of resilient coping, positive psychosocial strength, and proactive behaviors during public health crises [18,19]. Our results suggest that interventions that include strategies to enhance resilient coping and build hope such as community-wide mindfulness-based stress reduction training [34], web-based programs to promote positive psychology [35] and use of social media to enhance social connectedness and support [36], may be beneficial and help prevent mental health symptoms [33].

### Limitations

This study has several limitations. First, this study surveyed a convenience sample and was limited to those with Internet access; our sample also included more females and younger age participants and therefore may not represent a country’s general population. The results may suffer from additional selection bias because study participants may be more interested in the topic than those who did not participate. Second, self-reported personal COVID-19 exposure and mental health symptoms during the lockdown may have been affected by participants’ social desirability bias, which may result in either over- or under-estimations of mental health symptoms. Third, our results may have been affected by recall bias because some countries had already lifted their lockdown restrictions at the time of data collection. Fourth, a GSI score was not available for North Macedonia. Thus, we used the average scores of its five neighboring countries (Albania, Bulgaria, Greece, Kosovo, Serbia) instead, which may have been either under- or over-estimated. Finally, we only included 11 countries in this study, limiting our study’s ability to detect country-level effects on mental health outcomes.

## 5. Conclusions

The COVID-19 pandemic has resulted in substantial global mental health challenges, such as increased levels of anxiety and depression symptoms [4,24]. Our results suggest significant variations in residents’ anxiety and depression symptoms across countries. While personal COVID-19 exposure was significantly associated with anxiety and depression symptoms, country-level COVID-19 factors were not. Our results have important implications for future prospective cohort studies and multi-country interventions [33,34]. Future research should include more representative samples and multiple follow-ups to further our understanding of the short- and long-term effects of the COVID-19 lockdown on the mental health of the global population. Future studies should also develop population-based, culturally appropriate interventions that promote positive mental health and mitigate risk exposures in order to reduce the mental health burden during this and other global emergencies [34,35,36].

## Figures and Tables

**Table 1:** Demographic characteristics and COVID-19 exposure of participants in the 11 participating countries, *n* = 13,263 participants.

		*n*	%
Country			
	China	2850	21.5
	Brazil	1500	11.3
	Turkey	1400	10.6
	United States	1027	7.7
	Singapore	997	7.5
	Spain	981	7.4
	Ireland	980	7.4
	Bulgaria	952	7.2
	India	935	7.1
	Malaysia	831	6.3
	North Macedonia	810	6.1
Sex			
	Female	8332	62.8
	Male	4801	36.2
	Other	130	1.0
Age ^a^			
	18–24	4072	30.7
	25–34	2785	21.0
	35–44	2624	19.8
	45–54	1949	14.7
	55–64	1195	9.0
	65 years or older	625	4.7
Education ^a^			
	Less than a high school degree	873	6.6
	High school degree	2321	17.5
	Associate degree	2043	15.4
	Bachelor’s degree	4744	35.8
	Graduate degree	3258	24.6
Marital status			
	Single	6465	48.7
	Married	5858	44.2
	Other	885	6.7
History of a mental disorder			
	Yes	2022	15.3
	No	11,241	84.8
Number of people co-habiting during the lockdown ^a^			
	0	1891	14.3
	1	2351	17.8
	2	3252	24.6
	3	2941	22.2
	4+	2794	21.1
Employment and income changes since COVID-19 ^b^			
	Employment changed and income decreased	1135	8.6
	Employment changed but no change in income	559	4.2
	No change in employment but income decreased	2122	16.0
	No changes in employment nor income	4904	37.0
	Other	4543	34.3
Personal COVID-19 exposure ^c^			
	Went out less than once a month	1075	8.1
	Went out at least once a month	1351	10.2
	Went out at least once a week	5991	45.2
	Had a contact with COVID-19 patient or had to quarantine for 14 days	2939	22.2
	Diagnosed with COVID-19 or experienced COVID-19 symptoms	1907	14.4

Note: ^a^ Thirteen participants with a missing value for “Age”, 24 with a missing value for “Education”, 34 with a missing value for “Number of people co-habiting during the lockdown” were not included. ^b^ Variable created based on participants’ responses to two variables: changes in income and changes in employment status since the start of COVID-19 pandemic. ^c^ Variable created based on participants’ COVID-19-related exposures during their country’s lockdown, capturing their highest risk exposure in 1 of 5 mutually exclusive categories (5 = high-risk exposure, 1 = low-risk exposure).

**Table 2:** Zero-order correlation between country-level COVID-19 factors, personal COVID-19 exposure, and mental health outcomes. among participants in the 11 countries.

	Governmental Policy Responses	Personal COVID-19 Exposure		Anxiety	Depression	Resilient Coping	Hope
Increase in confirmed cases per million people ^a^	0.087 **	0.175 **		0.311 **	0.255 **	−0.068 **	−0.048
Governmental policy response ^a^		0.030 **		0.152 **	0.135 **	−0.099 **	−0.121
Personal COVID-19 exposure ^b^				0.144 **	0.132 **	−0.021 *	−0.070
Anxiety					0.765 **	−0.254 **	−0.344
Depression						−0.307 **	−0.449
Resilient coping							0.601

Note: * *p* value < 0.05; ** *p* value < 0.001, based on Pearson Correlation Coefficient tests. ^a^ Country-level COVID-19 factors were measured using two variables: *Increase in confirmed cases per million people,* capturing each participating country’s average confirmed COVID-19 cases per million people per day during their lockdown; and *Governmental policy response* to COVID-19, capturing each participating country’s average government stringency index score during their lockdown (0 = no policy response, 100 = strictest policy responses). Government stringency index score was not available for North Macedonia, thus, we used the average scores of its 5 neighboring countries (Albania, Bulgaria, Greece, Kosovo, Serbia) instead. ^b^ Personal COVID-19 exposure was created based on participants’ COVID-19-related exposures during their country’s lockdown, capturing their highest risk exposure in 1 of 5 mutually exclusive categories (5 = high-risk exposure, 1 = low-risk exposure).

**Table 3:** Associations of country-level COVID-19 factors and personal COVID-19 exposure with anxiety and depression among participants in the 11 countries.

	Anxiety			Depression		
	Model 2 (*n* = 12,671) β (95%CI)	Model 3 (*n* = 12,583) β (95%CI)	Model 4 (*n* = 12,524) β (95%CI)	Model 2 (*n* = 12,951) β (95%CI)	Model 3 (*n* = 12,872) β (95%CI)	Model 4 (*n* = 12,824) β (95%CI)
**Country-level COVID-19 factors**						
Increase in confirmed cases per million people	0.06 (0.00, 0.13)	0.06(0.00, 0.12) *	0.05 (0.00, 0.10)	0.05 (0.00, 0.11)	0.05(0.00, 0.10)	0.04 (−0.01, 0.10)
Governmental policy response	0.21 (−0.08, 0.51)	0.16 (−0.09, 0.41)	0.13 (−0.09, 0.36)	0.17 (−0.09, 0.42)	0.11 (−0.12, 0.34)	0.07 (−0.16, 0.30)
**Personal COVID-19 exposure**						
Went out less than once a month	Reference	Reference	Reference	Reference	Reference	Reference
Went out at least once a month	0.41 (−0.26, 1.08)	0.69 (0.09, 1.30) *	0.59 (−0.01, 1.17)	0.86 (0.15, 1.57) *	1.34 (0.71, 1.97) **	1.40 (0.79, 2.01) **
Went out at least once a week	0.00 (−0.56, 0.55)	0.57 (0.07, 1.07) *	0.65 (0.15, 1.14) *	−0.16 (−0.75, 0.43)	0.66 (0.14, 1.18) **	1.03 (0.52, 1.54) **
Had contact with a COVID-19 patient or had to quarantine for14 days	1.16 (0.57, 1.74) **	1.34 (0.81, 1.87) **	1.35 (0.83, 1.86) **	1.09 (0.47, 1.71) **	1.34 (0.80, 1.90) **	1.33 (0.79, 1.86) **
Diagnosed with COVID-19 or experienced COVID-19 symptoms	3.02 (2.37, 3.66) **	2.49 (1.91, 3.07) **	2.18 (1.61, 2.75) **	2.74 (2.06, 3.42) **	2.29 (1.69, 2.89) **	2.16 (1.57, 2.75) **
**Resilient coping**		−0.15 (−0.20, −0.10) **	−0.13 (−0.18, −0.08) **		−0.06 (−0.11, 0.00) *	−0.03 (−0.09, 0.02)
**Hope**		−0.56 (−0.59, −0.54) **	−0.54 (−0.57, −0.51) **		−0.67 (−0.69, −0.64) **	−0.61 (−0.64, −0.58) **
**Demographic variables**						
**Sex** ^a^						
Female			2.31 (2.04, 2.58) **			1.81 (1.54, 2.09) **
Male			Reference			Reference
**Age**						
18–24			2.78 (2.03, 3.53) **			3.33 (2.56, 4.10) **
25–34			2.46 (1.76, 3.16) **			2.74 (2.01, 3.46) **
35–44			1.95 (1.28, 2.62) **			1.96 (1.27, 2.66) **
45–54			1.35 (0.67, 2.02) **			1.19 (0.49, 1.89) **
55–64			0.82 (0.09, 1.54) *			0.46 (−0.29, 1.21)
65 years or older			Reference			Reference
**Education**						
Less than a high school degree			Reference			Reference
High school degree			−0.42 (−1.04, 0.21)			−0.88 (−1.52, −0.23) **
Associate degree			−0.45 (−1.05, 0.16)			−1.57 (−2.20, −0.94) **
Bachelor’s degree			0.35 (−0.23, 0.92)			−1.07 (−1.66, −0.47) **
Graduate degree			0.95 (0.33, 1.56) **			−1.05 (−1.68, −0.42) **
**Marital status** ^a^						
Single			−0.60 (−0.98, −0.23) **			−0.04 (−0.43, 0.35)
Married			Reference			Reference
**History of a mental disorder**						
Yes			3.04 (2.66, 3.42) **			3.62 (3.22, 4.01) **
No			Reference			Reference
**Employment and income changes since COVID-19** ^a,b^						
Employment changed and income decreased			1.39 (0.91, 1.86) **			1.89 (1.40, 2.38) **
No change in employment but income decreased			0.39 (0.01, 0.76) *			0.87 (0.48, 1.26) **
Employment changed but no change in income			0.77 (0.13, 1.41) *			1.19 (0.53, 1.85) **
No changes in employment status nor income			Reference			Reference
**Number of people co-habiting during the lockdown**			0.33 (0.25, 0.42) **			0.10 (0.01, 0.19) *
ICC ^b^ Country	0.140	0.1299	0.1125	0.096	0.104	0.105
AIC ^b^	89,000.8	85,665.1	84,456.0	92,701.8	88,861.0	87,683.4
BIC ^b^	89,001.6	85,665.9	84,456.8	92,702.6	88,861.8	87,684.2

Note: * *p* value < 0.05; ** *p* value < 0.001. ICC: Intra-level correlation coefficient; AIC: Akaike information criterion; BIC: Bayesian information criterion; CI: Confidence intervals. ^a^ Estimates for participants that reported “Other” on variables of sex, marital status, and employment and income changes since COVID-19 were not listed. ^b^ Variable created based on participants’ responses to two variables: changes in income and changes in employment status since the start of COVID-19 pandemic. ^c^ Model 1, not listed, was a null model with no fixed factors but a random intercept of the Country variable. For anxiety, null model *n* = 12,671, ICC Country = 0.231, AIC = 89,192.60, and BIC = 89,193.40; For depression, null model *n* = 12,951, ICC country = 0.155, AIC = 92,861.20, and BIC = 92,862.00. For Model 2, we added country-level COVID-19 factors and personal COVID-19 exposure variables as fixed factors. For Model 3, we added resilient coping and hope. Model 4 adjusted for demographic variables, including sex, age, educational level, marital status, employment and/or income changes since the start of the COVID-19 pandemic, the number of people co-habiting during lockdown, and history of a mental disorder.

## Data Availability

Anonymized data used and/or analyzed during the current study, along with detailed study protocol, are available from the corresponding authors on reasonable request. The data are not publicly available due to privacy restrictions.

## Global Community Health–COVID-19 Collaborative Research Team

**Brazil** (Carolin Lusby, Nara R.C. Oliveira, Fillipe S. Romano, William F. Santana, Giselle H. Tavares), **Bulgaria** (Daniela S. Dasheva, Alixander J. Rocha), **China** (Yuwei Cai, Jiaai Huang, Rashid Menhas, Xuehui Sang, Yu Weng), **India** (Komal Chandiramani, Nudrat Jahan, Meena Osmany, Sonia), **Ireland** (Ursula Kenny), North **Macedonia** (Mishko Djidrov, Gordana Miteva, Katerina Mitevska Petrusheva, Snezana Jovanova Mitkovska ), **Malaysia** (Nur Syamsina Ahmad, Mohd Rahimi Che Jusoh, Yee Cheng Kueh, Ayu Suzailiana Muhamad, Marilyn Li Yin Ong), **Singapore** (Peggy Boey, Taufiq I. Jamaludin, Yew Cheo Ng, Edward J. Say, Chea Nuan Yow), **Spain** (Maria Gloría Gallego-Jiménez, Carina Soledad González-González, Myriam Guerra-Balic, Sarah Muñoz-Violant, Manuel J Rodriguez-Allue), **Turkey** (Bijen Filiz, Bengu Guven, Ezel Nur Korur, Funda Coskun Ozyol, Yilmaz Yuksel), and **United States** (Laura M. Dattner, Amanda K. Hautmann).
